# A study of interleukin 1β levels in peri-miniscrew crevicular fluid (PMCF)

**DOI:** 10.1186/s40510-014-0030-4

**Published:** 2014-04-01

**Authors:** Nitika Monga, Sushma Chaurasia, Om Prakash Kharbanda, Ritu Duggal, Moganty Raja Rajeswari

**Affiliations:** 1 Division of Orthodontics and Dentofacial Deformities, Centre for Dental Education and Research, All India Institute of Medical Sciences, New Delhi 110029, India; 2 Department of Biochemistry, All India Institute of Medical Sciences, New Delhi 110029, India

**Keywords:** IL-1β, Miniscrew, Peri-implantitis, Biomarker, Cytokine

## Abstract

**Background:**

This study provides a vital insight in assessing the clinical and biochemical changes in interleukin (IL)-1β levels in peri-miniscrew crevicular fluid (PMCF) during the course of orthodontic tooth movement.

**Methods:**

The study comprised the analysis of IL-1β in peri-miniscrew crevicular fluid obtained from crevices around the miniscrews inserted in 11 patients (eight females and three males, mean age 17.3 ± 4.64 years) with all first premolar extraction and maximum anchorage requirement using miniscrew-supported anchorage. Miniscrews were loaded at 3 weeks after placement by 200-g nitinol closed coil springs of 9-mm length for en masse retraction. Peri-miniscrew crevicular fluid was collected at miniscrew placement (T1), at 3 weeks (T2/baseline) and on loading at 0 (T3) and 1 day (T4), 21 (T5), 72 (T6), 120 (T7), 180 (T8) and 300 (T9) days. IL-1β levels were estimated by enzyme-linked immunosorbent assay (ELISA). Peri-miniscrew tissue was examined for signs of inflammation, and also, miniscrew mobility was assessed with Periotest and handles of two mouth mirrors.

**Results:**

IL-1β levels in all miniscrews were significantly higher at T1 and peaked again at T4 showing a bimodal peak. However, there was a gradual and statistically significant decrease in IL-1β till T5, while further changes till the end of the study were statistically not significant.

**Conclusions:**

The changing levels of IL-1β levels in PMCF over a duration of 300 days are suggestive of the underlying inflammatory process. IL-1β levels in PMCF show a significant rise during miniscrew insertion and on immediate loading. The trend of gradually reducing IL-1β levels around the miniscrew over the period after loading towards baseline is suggestive of adaptive bone response to stimulus.

## Background

Miniscrews have drawn immense consideration in orthodontic armamentarium since they were initially used as contrivance for reinforcing orthodontic anchorage. These implements now stand poised to become one of the most imperative and useful means of orthodontic anchorage for eclectic clinical applications due to their ease in placement at various locations in the mouth. However, it is quite true that there is still some apprehension regarding their stability and hence usefulness in providing absolute orthodontic anchorage during the entire course of treatment.

The reported success rate of the miniscrews ranged from 71.4% to 100% [1,2]. Peri-implantitis accounts for about 30% of miniscrew failures [3]. Peri-implantitis is a progressive peri-implant bone loss in conjunction with soft tissue inflammatory lesion. Initially, peri-mucositis (a reversible inflammation of the soft tissues surrounding the miniscrew) occurs, which if left untreated may progress to peri-implantitis [4]. An early and reliable detection of any adverse peri-miniscrew tissue reaction is essential for patients being treated with miniscrew [5].

Recent studies show that in periodontal and peri-miniscrew tissues, cytokines, tumour necrosis factor and transforming growth factor have important roles in regulating and amplifying inflammatory response [5-7]. Interleukin (IL)-1β is one of the most potent cytokine in the inflammatory process in the oral cavity triggered by various stimuli including neurotransmitters, bacterial products, other cytokines and mechanical forces. The action of IL-1β includes attracting leucocytes and stimulating fibroblasts, endothelial cells, osteoclasts and osteoblasts to promote bone resorption and to inhibit bone formation [8].

Peri-miniscrew crevicular fluid (PMCF) is an osmotically mediated inflammatory exudate originating from the vessels of the gingival plexus. Its composition is similar to that of the gingival crevicular fluid (GCF) containing host-derived enzymes and their inhibitors, inflammatory mediators, host response modifiers and tissue breakdown products [9]. Analysis of PMCF offers a non-invasive means of studying host response in peri-miniscrew disease and may provide an early indication of patients at risk for active disease [10].

There are several reports on IL-1β in dental implants; however, IL-1β in PMCF around miniscrews has not been explored enough. Sari and Ucar [7] studied the level of IL-1β around miniscrews starting 2 weeks after miniscrew placement and activation of coil spring, followed up to 21 days. No study has been reported so far about the changes in IL-1β levels at miniscrew placement, before loading and after loading during the course of extraction space closure. Therefore, this comprehensive study was undertaken with the aim to evaluate the changes in IL-1β levels in PMCF at miniscrew placement, before and after application of orthodontic forces and its correlation with the clinical parameters over the course of orthodontic treatment.

## Methods

PMCF analysis was conducted from samples collected around the crevices of miniscrews placed in 11 bimaxillary protrusive patients (mean age 17.3 ± 4.64 years) requiring all first premolar extraction and treated with Roth prescription (0.022″ × 0.028″). The patients were followed for 300 days. In one of the patients, two miniscrews failed 2 weeks after placement; hence, the patient could not be followed throughout the period of the study. Therefore, the study was conducted to follow a total of 40 miniscrews in 10 patients for 300 days. Ethical clearance was obtained from the Ethics Committee of All India Institute of Medical Sciences, New Delhi with letter reference number IESC/T-20/29.01.10 dated 8 March 2010 and letter reference number IESC/T-22/2011 dated 13 January 2011. Written consent was obtained from each patient prior to their inclusion in the study. None of the patients had history of any systemic disease, hormonal imbalance or drug intake, and all had satisfactory periodontal health prior to the start of orthodontic treatment.

**Figure 1:**
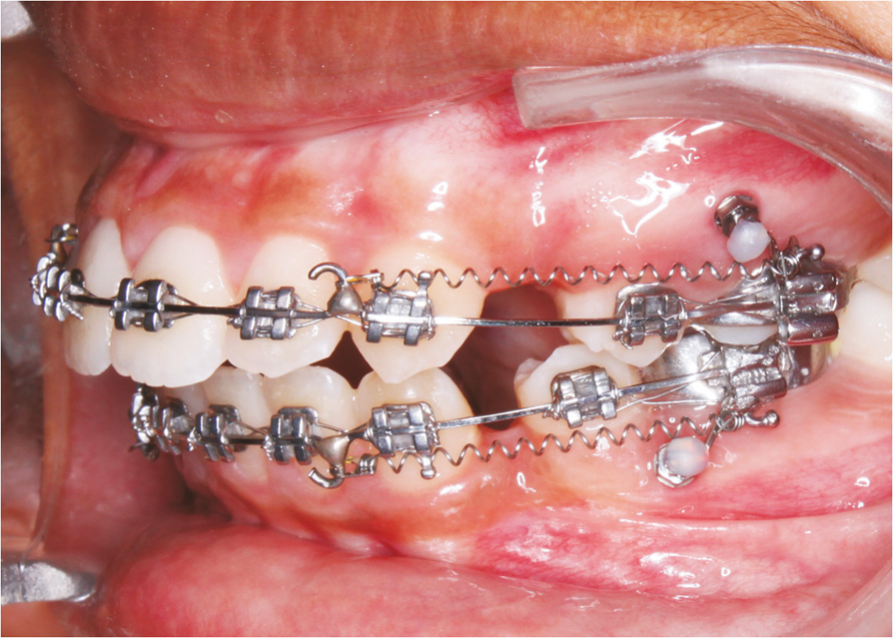
Assembly showing retraction mechanics using miniscrew as anchorage.

The buccal interradicular bone in the attached gingiva between the second premolar and first molar was chosen as site for miniscrew placement. Miniscrews (8-mm long, 1.5 mm in diameter, bracket head type; Absoanchor, Dentos, Daegu, South Korea) were placed at the intended sites at 60° to the long axis of the teeth under local anaesthesia [11]. Miniscrews were connected to the first molars using a 0.017″ × 0.025″ stainless steel wire passively, thereby providing indirect anchorage. En masse retraction was initiated 3 weeks after miniscrew insertion with a 9.0-mm nitinol closed coil spring (200-g force) attached to the soldered hook in between the lateral incisor and canine in a 0.019″ × 0.025″ stainless steel wire in both the arches. The retraction appliance was adjusted so as to provide constant force till space closure was achieved. However, the amount of force that was loaded to the miniscrew head at T3 and continued up to T9 might be different from 200-g because a sectional assembly was connected between the implant head and accessory buccal tube on the first molar (Figure 1).

The patients were instructed and followed for oral hygiene, more so around the peri-miniscrew with frequent brushing using a soft brush and mouth rinsing with 0.5 oz of 0.2% chlorhexidine gluconate twice daily. No anti-inflammatory drug was prescribed prior to and after miniscrew placement.

### Schedule of sample collection and clinical examination

Each sample of PMCF was collected by a 1 to 5-μl calibrated volumetric micro-capillary pipette (Sigma Aldrich Chemicals Company Limited, Bangalore, India) placed at the crevice, according to the method described by Griffiths [12]. The samples were collected at the following time observations: T1 (4 h after miniscrew placement), T2 (baseline, 3 weeks just prior to loading) and on loading T3 (0 day), T4 (1 day), T5 (21 days), T6 (72 days), T7 (120 days), T8 (180 days) and T9 (300 days) (Table 1). Although PMCF sample T1 was taken 4 h after miniscrew placement to allow cessation of bleeding, PMCF samples that were contaminated with the remaining blood during miniscrew placement procedure at T1 were discarded. Isolation of the miniscrew site was done with a cheek retractor, cotton rolls and a gentle air spray. PMCF (1 μl) was collected in Eppendorf tubes for 5 to 20 min from each test site at 25°C. Implant placement and PMCF sample collection was done in the morning hours between 9 a.m. and 11 a.m. except for T1 which was collected between 1 p.m. and 3 p.m. since it had to be collected 4 h after implant placement. GCF samples were gently collected, and extreme care was taken not to cause any slightest harm or injury during the sample collection. The samples thus obtained were diluted to 1:100 with Sorensen's buffer (0.05% bovine serum albumin in phosphate buffered saline, ph 7.0), centrifuged at 2,000 rpm for 1 min and stored at −70°C until assayed for interleukin levels.

**Table 1:** Schedule of PMCF sample collection and clinical examination

**Time**		**Schedule of sample collection**
Before loading	T1	Miniscrew placement
	T2	3 weeks prior to loading
After loading	T3	Immediately after loading
	T4	1 day after loading
	T5	At 21 days
	T6	At 72 days
	T7	At 120 days
	T8	At 180 days
	T9	At 300 days

IL-1β levels were estimated using human IL-1β ELISA kit Diaclone-Eli-pair (Besancon, France). Optical density was measured at 450 nm using Omega Microplate Reader™ (BMG Labtech, Ortenberg, Germany). The quantification of IL-1β was determined using standard curves for IL-1β, which showed a direct relationship between optical density and cytokine concentration.

Presence of any signs of peri-miniscrew inflammation (redness, swelling, pain) was detected clinically. Miniscrew mobility was assessed by a single operator using Periotest™ (Bensheim, Germany) with values of 0 to 9 on the Periotest scale indicating that the miniscrew is mechanically stable and values above 9 indicating clinical failure, and blunt ends of mouth mirror handles were used for visual evaluation [13]. A miniscrew was considered healthy if there was no visible sign of inflammation with no or physiologic mobility (Periotest value ranging from 0 to 9). Presence of mobility (visual miniscrew mobility scales 1 to 3, Periotest value more than 9) was considered the distinguishing feature between mucositis and peri-implantitis (Table 2).

**Table 2:** Scale for assessment of peri-miniscrew tissue health and mobility

	**Clinical signs**	**Mobility**	
		**Clinical miniscrew mobility scale**	**Periotest value**
Healthy	No redness, swelling, pain	No detectable mobility (0)	<9
Mucositis	Redness, swelling, pain present	No detectable mobility (0)	<9
Peri-implantitis	Redness, swelling, pain present	Detectable mobility (1 to 3)	>9

### Statistical analysis

Data was analyzed using SPSS version 20 and represented in median, maximum and minimum. Changes in between the quadrants were analyzed using the Friedman test followed by multiple comparisons using Wilcoxon signed-rank non-parametric test with Bonferroni correction. A *p* value <0.05 was taken as statistically significant, and in multiple comparison, a *p* value is considered significant after Bonferroni test.

## Results and discussion

### Results

**Figure 2:**
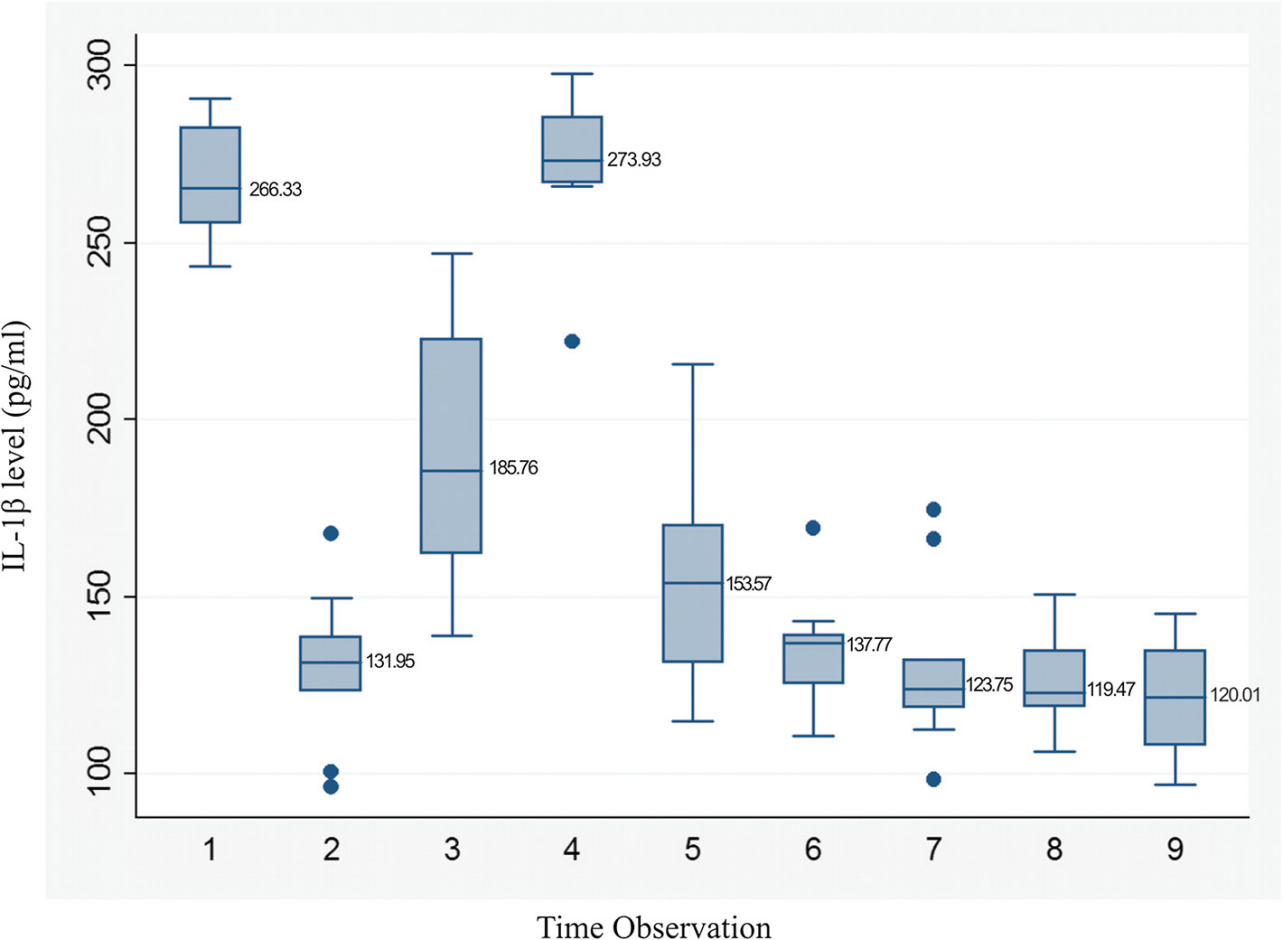
**Trend of IL-1β levels in PMCF at different time intervals.** 1-T1 (4 hours after Implant placement), 2-T2 (Baseline: 3 weeks, just prior to loading), 3-T3 (Just after loading: 0 day), 4-T4 (At 24 hours after loading: 1 day), 5-T5 (At 21 days), 6-T6 (At 72 days), 7-T7 (At 120 days), 8-T8 (At 180 days) and 9-T9 (At 300 days). The boxes represent the values from the 25th to the 75th percentile. The middle lines represent the medians. The vertical lines extend from the minimal to the maximal values, exluding the outliers marked with small dark circles.

The levels of IL-1β at each site were first tabulated and compared for each quadrant in each patient. The trend was found to be the same with no statistically significant difference among the four quadrants. Figure 2 shows the average taken for all four quadrants of ten patients followed for 300 days.

IL-1β level at T1 was 266.33 pg/ml which was significantly higher as compared to that at baseline (T2) being 131.95 pg/ml (*p* = 0.001). The levels rose slightly to185.76 pg/ml at T3 compared to T2. At T4, the levels had risen significantly to 273.93 pg/ml (*p* = 0.001). The levels reduced to 153.57 pg/ml at T5 (*p* = 0.014) and further to 137.77 pg/ml at T6. The values were statistically significant till T5, following which the levels further declined but were not statistically significant (Table 3) with levels falling to 120.01 pg/ml at T9 (*p* = 0.737).

**Table 3:** Level of IL-1β (pg/ml; median) at various time observations

	**Median**	**Range**	***p*** **value against T2**
T1	266.33	243.27 to 290.58	0.001
T2 (baseline)	131.95	96.17 to 167.65	
T3	185.76	138.96 to 246.89	0.001
T4	273.93	222.10 to 297.56	0.001
T5	153.57	114.91 to 215.76	0.015
T6	137.77	110.61 to 169.34	0.391
T7	123.75	98.32 to 174.50	0.881
T8	119.47	106.14 to 150.72	0.794
T9	120.01	96.82 to 145.40	0.737

*p* value against T2 as baseline.

Three miniscrew sites were reported with mucositis initially at T2 and one site at T5 (Table 4). Periotest values for healthy as well as mucositis sites were between 0 and 9, and clinical miniscrew mobility scale was 0 with no clinically detectable mobility although miniscrews with mucositis did show variable amount of redness, swelling and/or pain. Peri-implantitis was reported in two sites in the same patient, the right and left sides of the mandible, at 2 weeks after placement, which was excluded from the study. IL-1β levels at T1 in these two sites were 274.83 and 228.13 pg/ml, respectively (Table 5). At miniscrew failure, the level of IL-1β rose significantly to 361.63 and 385.35 pg/ml, respectively, as against two stable miniscrews in the maxilla in the same patient where the level of IL-1β decreased to 93.71 and 103.24 pg/ml, respectively, at 2 weeks after placement, from 167.41 and 192.67 pg/ml, respectively, at T1. Two miniscrews with peri-implantitis had Periotest values of 22 and 29, respectively, and the clinical miniscrew mobility scale was 3 with mobility evident. Both the sites clinically showed redness and swelling, but the patient did not complain of pain.

**Table 4:** Trend in the levels of IL-1β (pg/ml) in mucositis patients

**Case number**	**Affected quadrant**	**Unaffected quadrant**	**Observation time**								
			**T1**	**T2**	**T3**	**T4**	**T5**	**T6**	**T7**	**T8**	**T9**
2	I	II, III, IV	104.7	171.1^a^	206.6^a^	269.1^a^	187.6	193	136.6	173.4	132.5
4	II	I, III, IV	277.2	110.1	168	193.8	226.6^a^	233.2^a^	123	147.3	135.2
8	III	I, II, IV	329.9	247.8^a^	247^a^	269.1^a^	242.2	183.7	107.6	113.1	148.6
9	III	I, II, IV	322.7	244.8^a^	301^a^	338.6^a^	244.8^a^	244.8^a^	197.8^a^	167.5^a^	125.7

One mucositis site was found in both quadrants I and II and two in quadrant III. Quadrant I, right maxilla; quadrant II, left maxilla; quadrant III, left mandible; quadrant IV, right mandible. ^a^Presence of mucositis in the above mentioned quadrants.

**Table 5:** Trend in the levels of IL-1β in pg/ml in a peri-implantitis patient

**Quadrant**	**Observation time**					
	**T1**	**T2**	**T3**	**T4**	**T5**	**T6**
I	167.41	93.71	139.17	284.42	172.05	102.05
II	192.67	103.24	130.17	292.98	191.23	162.88
III	274.84	361.63^a^	*Failure*			
IV	228.13	385.35^a^				

Quadrant I, right maxilla; quadrant II, left maxilla; quadrant III, left mandible; quadrant IV, right mandible. ^a^Presence of peri-implantitis in the above mentioned quadrants.

### Discussion

Miniscrew success has been recognized as the ability of screw to sustain anchorage loads during orthodontic treatment without inflammation and mobility. Various studies present in the literature attempt to assess the stability of miniscrews from different perspectives such as clinical [3,14,15], biomechanical [16-18], histological [15],[19-21], or biochemical. Several biochemical markers have been associated with inflammation and remodelling of peri-implant tissue around osseo-integrated dental implants [20-23], but only few studies were found in the literature in which biochemical assessments were used to investigate the stability of miniscrews [7],[24-26]. In the study by Sari and Ucar [7], IL-1β levels in PMCF were collected only after loading to determine the effects of mechanical stress on the miniscrews. The mean IL-1β level in PMCF of healthy miniscrews did not increase significantly during orthodontic loading. Intachai et al. [24] assessed medians of chondroitin sulphate levels in PMCF around ‘immobile’ miniscrews, between the unloaded and loaded periods, which were not significantly different.

In our prospective study, IL-1β levels were observed at various times, both before and after the loading of miniscrew. It was started with the belief that the clinical stability of the miniscrew must reflect the activity of the underlying biomarkers.

In the present study, the first PMCF sample was collected 4 h after miniscrew placement, allowing cessation of bleeding. The early acute inflammatory changes account for junctional retraction that occurred in response to the cytokine (IL-1β) which begins within 4 to 6 h after injury and lasts for more than 24 h due to the trauma during the miniscrew placement procedure. Further, IL-1 elicits the release of histamine from mast cells at the site of inflammation which then triggers early vasodilatation and increased vascular permeability [27].

The fall of IL-1β level at T2 was observed at 3 weeks whereby miniscrew remained unloaded during this period. This decreased level suggests that at 21 days, there was no more cellular stress in the absence of load. A slight increase in the IL-1β level after loading can be explained by the fact that in orthodontics, mechanical stress appears to evoke biochemical and structural responses in a variety of cells *in vivo* and *in vitro*[28]. Alhashimi et al. [29] showed that the application of orthodontic force induces *de novo* synthesis of proinflammatory cytokines IL-1β and IL-6, which support the hypothesis that these proinflammatory cytokines play important roles in bone resorption during the application of orthodontic force. IL-1 upregulates receptor activator of nuclear factor κ B ligand (RANKL) which leads to bone resorption [30]. Further, according to Kanzaki et al. [31], bone resorption is regulated by cytokines released in response to the orthodontic force.

Although there were two peaks of IL-1β levels during the study attributed to delayed loading, it was done to assess the change in IL-1β level around the unloaded miniscrew apart from observing the effect of loading on IL-1β levels. A 3-week period before loading was selected based on the study which states that loading during the first 2 weeks of healing time would damage the stability of the implant-bone fixture [32,33], further emphasizing on the loading period of 3 weeks.

Transient elevation of IL-1β 24 h after loading shows that the cells within the attached gingiva surrounding the miniscrew produce IL-1β in response to the orthodontic stimulus which can be detected in the PMCF. This level of IL-1β peaked since during acute inflammation, IL-1β which is released 1 to 2 h after mechanical stimulus peaks at 24 to 48 h [27]. Though IL-1β levels rose significantly, the miniscrews were stable. Huja et al. [34] also demonstrated that miniscrews could withstand significantly greater force levels and when loaded purely in an axial direction; they observed maximal force loads of 388.3 N.

Over the period, there was a gradual fall in IL-1β levels at 21 days after loading, and levels declined further over the 300-day study period reaching the baseline. This could be explained by the fact that cytokines are involved in extensive networks that involve synergistic as well as antagonistic interactions and exhibit both negative and positive regulatory effects on various target cells. IL-1 receptor antagonist (IL-1Ra) inhibits IL-1 action by competing with IL-1 to bind to the IL-1 receptor (IL-1R) [35,36]. This kind of feedback or negative regulation is likely to modulate the remodelling process *in vivo* after a synergistic or an additive effect because a number of cytokines are simultaneously involved in the mechanically activated periodontal cells. Also, this decrease might be attributed to the adaptation of periodontal tissues to the orthodontic force, and feedback mechanisms might prevent an excessive increase in the inflammation mediators, thereby preventing harmful consequences [37].

An important regulatory step in the expression of the cytokines is gene transcription. Several positive and negative transcription factors function in a concerted manner to regulate the transcription of cytokine genes to initiate immediately on activation and to shut down quickly, even in the continuous presence of the stimulating agent [38]. IL-1β levels in this study are high as compared to those of a previous study [7] which could probably be attributed to racial variation [39].

Mucositis was reported in three miniscrew sites 3 weeks after miniscrew placement and in one site 21 days after loading. This could be due to the poor oral hygiene at these sites. Plaque around miniscrews acts as a local factor for inflammation. Chemoattractant effect of bacterial lipopolysaccharide present in plaque activates macrophages and polymorphonuclear leukocytes to produce inflammatory mediators like TNF-α, IL-1, IL-6 and other cytokines related to host response and tissue destruction [40]. With regular oral hygiene instructions and professional oral hygiene measures, the peri-miniscrew inflammation subsided.

Peri-implantitis was reported in two miniscrews 2 weeks after placement, before loading, both in the mandible of the same patient. It suggests that the primary factor causing miniscrew loss is peri-miniscrew soft tissue inflammation, not the application of orthodontic force. A further indication that low-magnitude static forces are not detrimental to miniscrew success was presented in a study [41], in which miniscrew losses were attributed to infection and occurred during healing periods, not after load activation. The levels of IL-1β from the failed miniscrews were high even at the time of miniscrew placement. The elevation of IL-1β levels before miniscrew failure might be attributed to underlying bone resorption around the miniscrew, but the sample size of peri-implantitis is too small to draw conclusions.

Animal and human studies have shown that the levels of sex steroids, including progesterone and oestradiol, fluctuate in accordance with oestrous or menstrual cycle. Osteoclast activity can be inhibited by oestrogen in a direct or indirect manner, thereby modulating bone resorption [42]. This aspect is beyond the scope of this study since it focused on changes in IL-1β levels with time and also due to the unequal and small sample size with a male/female ratio of 7:3.

In this study, PMCF sample collection was done 3 weeks after miniscrew placement. The authors believe that more frequent sample collection could have been done during this 3-week period and results of this study could be further confirmed with a larger sample size. Further, miniscrews were indirectly loaded; thereby, precise load on miniscrew cannot be calculated. Since IL-1β levels rise both during inflammation as well as during orthodontic loading, the IL-1B levels in PMCF may only represent the release of cytokine as an inflammatory response but may not represent the bone stress from orthodontic loading especially at T6 to T9. Thus, alveolar bone remodelling biomarkers, in addition to inflammatory mediators, are suggested to be monitored during further investigations.

## Conclusions

The changing levels of IL-1β in PMCF over a duration of 300 days are suggestive of the underlying inflammatory process. IL-1β levels in PMCF show a significant rise during miniscrew insertion and on immediate loading. The trend of gradually reducing IL-1β levels around the mini-screw over the period after loading towards the baseline is suggestive of adaptive bone response to stimulus.

## Abbreviations

IL-1β: Interleukin 1β: 

PMCF: Peri-miniscrew crevicular fluid: 

GCF: Gingival crevicular fluid: 

## Competing interests

The authors declare that they have no competing interests.

## Authors’ contributions

NM started the initial seven cases and followed the cases up to 72 days. SC followed the same cases up to 300 days and, in addition, completed three more cases. OPK and RD conceived and supervised the study, participated in its design and coordination and helped draft the manuscript. RMR helped with all biochemical assays. All authors read and approved the final manuscript.
